# 

**Published:** 1879-09

**Authors:** 


THE demand for copies of the initial number of the present volume of the JOURNAL, has been so greatly in excess of our expectations, that we have been compelled to refuse many applications. The present edition is nearly double that of the previous volume. We hope to supply all demands for specimen copies of this number. Subscribers desiring a complete file of the present volume, must make early application for the August issue.

With this number we enclose bills for the current year, and we ask a prompt response. It is our aim to make the JOURNAL a necessity to every educated physician in Western New York, and also a welcome visitor to the profession in other sections. But we must remind our patrons that the subscription price given has been reduced to so low a figure that the JOURNAL can be successfully conducted only upon the *cash principle.* We are especially gratified with the favorable reception, so generously bestowed by the profession upon our August number. The present and future numbers will deserve, we trust, a yet more favorable opinion.

